# Complete genome sequences of *Streptococcus agalactiae* urine isolates collected from postmenopausal women

**DOI:** 10.1128/mra.00578-26

**Published:** 2026-07-23

**Authors:** Nikki S. Koonjbearry, Michael L. Neugent, Belle M. Sharon, Philippe E. Zimmern, Nicole J. De Nisco

**Affiliations:** 1 Department of Biological Sciences, The University of Texas at Dallas 12335, Richardson, Texas, USA; 2 Department of Urology, University of Rochester Medical Center 6923, Rochester, New York, USA; 3 Department of Urology, The University of Texas Southwestern Medical Center 12334, Dallas, Texas, USA; University of Pittsburgh School of Medicine, Pittsburgh, Pennsylvania, USA

**Keywords:** group B* Streptococcus*, urinary tract infection, human microbiota, genomes

## Abstract

We report complete genome sequences of two *Streptococcus agalactiae* isolates recovered from the urine of postmenopausal women with a history of recurrent urinary tract infection. These high-quality genomes will support ongoing efforts to understand *S. agalactiae* strain-level genetic variation and its potential role in urinary tract disease in this population.

## ANNOUNCEMENT

*Streptococcus agalactiae*, also known as Group B *Streptococcus* (GBS), is a gram-positive bacterium that colonizes the gastrointestinal and genital tracts of healthy adults (1). Although typically harmless, *S. agalactiae* can cause infection in at-risk populations, especially in elderly or pregnant people, and is the leading cause of neonatal infections (2). GBS infections can be severe, especially in cases of neonatal meningitis and blood infection (1). Elderly women are also impacted as menopause-associated hormonal changes increase urogenital microbiome dysbiosis and urinary tract infection (UTI) risk (3). Urine culturomics has found significantly more *S. agalactiae* in women with UTI compared to controls (4). Overall, the prevalence of *S. agalactiae* in the female urogenital tract and its association with human disease highlight the need for expanded genomic characterization of this clinically important species.

We report the complete genome assemblies of two *S*. *agalactiae* urine isolates from postmenopausal women collected as part of Institutional Review Board-approved studies STU032016-006 (UT-Southwestern Medical Center) and 19MR0011 (UT-Dallas) in 2019. Midstream “clean-catch” urine was obtained from women with a documented history of recurrent UTI (rUTI). At collection, one patient had an active UTI (rUTI relapse), while the other was asymptomatic (rUTI remission) (5). Urine was plated onto sheep blood agar and incubated at 37°C microaerophilically using GasPak EZ Campy pouches (BD). Species identification was performed by Sanger sequencing of the PCR-amplified 16S ribosomal RNA gene using 8F and 1492R primers (6). Sanger sequencing results were analyzed with MegaBLAST (BLAST v2.10.0) (7). Genomic DNA was extracted from 18-h cultures grown statically in brain heart infusion broth at 37°C, ambient atmosphere using the Qiagen DNeasy blood and tissue kit. The DNA concentration and quality were assessed using 260/280 nm absorbance ratio and 0.8% agarose gel electrophoresis.

Long-read libraries were constructed using the Oxford Nanopore ligation sequencing kit (SQK-LSK109) and EXP-NBD104 barcodes. Sequencing was accomplished on a MinION utilizing R9 FLO-MIN106 flow cells, and data were processed by Oxford Nanopore Technologies (ONT) MinKNOW v21.05.21, where fast basecalling, barcode removal, and demultiplexing were performed in real time. Read quality was assessed with NanoFilt v2.6.0 (8). Reads shorter than 200 bp and a Phred score under 7 were removed using NanoStat v1.2.0 (8). Illumina libraries were constructed with the Nextera DNA Flex kit and sequenced using a NextSeq 500 to generate 2 × 150 bp paired-end reads. Sequences were quality checked using CLC Genomic Workbench v12.0.3, where reads <15 bp and Phred score below 20 were filtered out.

Unicycler v0.4.8 (9), SPAdes v3.13.0 (10), Racon v1.4.10 (11), and Pilon v1.2.3 (12) were used for generating hybrid assemblies. Each closed chromosome was rotated to begin at the *dnaA* gene. We evaluated assembly quality using QUAST v5.0.2 (13) and checked for completeness with the bacterial ortholog set from gVolante v1.2 (14). Closed genomes were verified using Bandage v0.8.1 (15) (14) and BUSCO v1 (16). Both genomes were identified as sequence type 459 by PubMLST (17). Geneious Prime v2020.0.5 was used to calculate GC content and identify coding sequences. Gene annotation was performed with the National Center for Biotechnology Information Prokaryotic Genome Annotation Pipeline v4.11 (18). Software default parameters were used for all steps unless specified otherwise.

**TABLE 1:** Accession numbers, assembly parameters, and isolate characteristics of two *Streptococcus agalactiae* strains isolated from the urine of postmenopausal women with a history of recurrent UTI

Strain	Host health state	BioSample accession no.	SRA accession no.^*b*^	No. of raw reads	No. of trimmed reads	Read *N*_50_ (bp)	Read depth (×)	GenBank accession no.	Type of contig (circular)	Total length (bp)	GC content (%)	No. of CDSs^*a*^
Sag 1327	rUTI Remission	SAMN53396779	SRX31920522 (I)	8,273,336	8,141,072		620	GCA_054847965.1	Chromosome	2,223,168	35.52	2,158
			SRX31920523 (O)	128,953	127,228	10,169	338					
Sag 1689	rUTI Relapse	SAMN53396780	SRX31920520 (I)	9,148,436	8,977,688		673	GCA_054847945.1	Chromosome	2,262,479	35.51	2,223
			SRX31920521 (O)	176,001	175,211	10,327	486					

^*a*^ Coding sequence.

^*b*^ Sequencing technology used is shown in parentheses. O, ONT; I, Illumina.

## Data Availability

These genome sequences are available in GenBank under BioProject accession number PRJNA1369612. Hyperlinked BioSample and SRA accession numbers can be found on Table 1.
